# Strain-Dependent Effects on Confinement of Folded Acoustic and Optical Phonons in Short-Period (XC)_m_/(YC)_n_ with X,Y (≡Si, Ge, Sn) Superlattices

**DOI:** 10.3390/ma17133082

**Published:** 2024-06-23

**Authors:** Devki N. Talwar, Sky Semone, Piotr Becla

**Affiliations:** 1 Department of Physics, University of North Florida, 1 UNF Drive, Jacksonville, FL 32224, USA; 2 Department of Physics, Indiana University of Pennsylvania, 975 Oakland Avenue, 56 Weyandt Hall, Indiana, PA 15705, USA; 3 Department of Electrical Engineering, The Pennsylvania State University, 207 Electrical Engineering West, University Park, PA 16802, USA; sys5834@psu.edu; 4 Department of Materials Science and Engineering, Massachusetts Institute of Technology, Cambridge, MA 02139, USA; becla@mit.edu

**Keywords:** novel C-based (XC)_m_/(YC)_n_ superlattices, interfacial layer thickness, Raman intensity profiles, lattice dynamics, localization of atomic displacements

## Abstract

Carbon-based novel low-dimensional XC/YC (with X, Y ≡ Si, Ge, and Sn) heterostructures have recently gained considerable scientific and technological interest in the design of electronic devices for energy transport use in extreme environments. Despite many efforts made to understand the structural, electronic, and vibrational properties of XC and X_x_Y_1−x_C alloys, no measurements exist for identifying the phonon characteristics of superlattices (SLs) by employing either an infrared and/or Raman scattering spectroscopy. In this work, we report the results of a systematic study to investigate the lattice dynamics of the ideal ${\left(XC\right)}_{m}$/${\left(YC\right)}_{n}$ as well as graded ${\left(XC\right)}_{10-∆}/{\left(X_{0.5}Y_{0.5}C\right)}_{∆}/{\left(YC\right)}_{10-∆}/{\left(X_{0.5}Y_{0.5}C\right)}_{∆}$ SLs by meticulously including the interfacial layer thickness ∆ (≡1–3 monolayers). While the folded acoustic phonons (FAPs) are calculated using a Rytov model, the confined optical modes (COMs) and FAPs are described by adopting a modified linear-chain model. Although the simulations of low-energy dispersions for the FAPs indicated no significant changes by increasing ∆, the results revealed, however, considerable “downward” shifts of high frequency COMs and “upward” shifts for the low energy optical modes. In the framework of a bond polarizability model, the calculated results of Raman scattering spectra for graded SLs are presented as a function of ∆. Special attention is paid to those modes in the middle of the frequency region, which offer strong contributions for enhancing the Raman intensity profiles. These simulated changes are linked to the localization of atomic displacements constrained either by the XC/YC or YC/XC unabrupt interfaces. We strongly feel that this study will encourage spectroscopists to perform Raman scattering measurements to check our theoretical conjectures.

## 1. Introduction

Since the pioneer work of Esaki and Tsu in 1969 [1], the experimental and theoretical studies on low-dimensional heterostructures (LDHs) [i.e., multi-quantum wells (MQWs) and superlattices (SLs)] have been the most exciting areas of research [2,3,4,5,6,7,8,9] among material scientists, solid-state engineers, and physicists [10,11,12,13,14,15,16,17,18,19,20,21,22,23,24,25,26,27,28,29,30,31,32,33,34,35,36,37,38,39]. Consistent advances in the epitaxial growth by molecular beam epitaxy (MBE) [10,11,12] and metal organic chemical vapor deposition (MOCVD) [13,14] techniques have made it possible to design high-quality MQWs and SLs. As LDHs do not exist in nature, one needs to prepare them epitaxially by combining two or more ultrathin layers of different materials choosing a discrete number of atomic layers (or thicknesses) and stacking them periodically. This process has offered many flexibilities for creating different device structures at the molecular level. In anticipation of the innovation in technology, two types of LDHs are envisioned: (a) compositional [15,16,17,18,19,20,21] and (b) doped [22,23]. The most attractive feature of SLs has been and still is to plan novel artificial structures with properties otherwise not available in bulk and/or alloyed materials. To explore the viability of LDHs, Esaki and Tsu [1,2,3,4,5,6,7,8] selected semiconductors with potential barriers and quantum wells thin enough to exhibit resonant electron tunneling [40,41,42,43,44,45,46,47]. This ingenuity [1,2,3,4,5,6,7,8] is now considered to be the greatest achievement in many insightful ways [48,49,50,51,52,53,54,55,56,57,58,59,60,61,62,63,64,65,66,67,68,69,70]. Successful productions of SL-based electronic, optical, optoelectronic, and magneto-optical devices have helped scientists and engineers to integrate them into different electronic circuits [71,72,73,74,75,76,77,78,79,80,81,82,83,84,85,86,87,88].

Historically, the most prominent lattice-matched SL ever matured is the (GaAs)_m_/(Ga_1−x_Al_x_As)_n_ [3,4], where m and n layers of GaAs, Ga_1−x_Al_x_As form quantum wells and potential barriers, respectively. Apart from the success of exploring basic traits of quantum phenomena in solid-state physics [11,12,13,14,15,16,17,18,19,20,21,22,23,24,25,26,27,28,29,30,31,32,33,34,35,36,37,38,39], these SLs have instigated many perspectives regarding technological applications [40,41,42,43,44,45,46,47]. By varying m, n, and/or composition x, the modifications of electronic, optical, thermal, phonon, and acoustic characteristics are used for realizing high-speed, high-mobility vertical transport, sequential tunneling, photonics, electro-optical modulators (EOMs), photo diodes (PDs), and infrared (IR) avalanche photodetectors (IR-APDs)—mostly for operations in the IR spectral region [48,49,50,51,52,53,54,55,56,57,58,59,60,61,62,63,64,65,66,67,68]. The unique concepts of LDHs have also been expanded to acquire other semiconductor devices with a functional quantum Hall effect, electron-phonon confinement, inter-sub-band emission, and tunneling phenomenon [40,41,42,43,44,45,46,47,48,49,50,51,52,53,54,55,56,57,58,59,60,61,62,63,64,65,66,67,68,69,70,71,72,73,74,75,76,77,78,79,80,81,82,83,84,85,86,87,88]. Despite several successes, there is a widespread reluctance among the epitaxial growth community to address the challenges in making devices for renewable energy, optical imaging, sensing, and detection needs. To improve SL-based devices with expanded functionalities and reduction in sizes, the use of III-Ns [89,90,91,92,93] and C-based IV-IV materials has recently [94,95,96,97,98,99,100,101,102,103] progressed in creating mid-infrared (MIR) devices for high-temperature electronics, healthcare, photovoltaic, and automotive industry requirements.

Despite earlier conceptual constraints of Si to generate light, the Si-centered optical platform has now rapidly changed the landscape of photonic integrated circuits (PICs) by offering powerful solutions to telecom, datacom, bio-photonics, and quantum networks [95,96,97,98,99,100,101,102,103,104,105,106,107,108,109,110,111,112,113,114,115,116,117,118,119,120]. With excellent optical properties of Si, many integrated passive and active devices are instigated due to the high refractive index contrast waveguides of Si on insulator (SOI). The exploration of novel materials with ultra-low loss and high electro-optic coefficients are examined to realize advanced PICs with monolithically integrated light sources and efficient modulators [94,95,96,97,98,99,100,101,102]. In this context, Si_1−x_Ge_x_ alloys are well established in the photonic and electronic industries [114]. Both Si and Ge are fully miscible across the entire composition x, enabling the tuning of basic properties, including lattice constant $a_{o}$ and bandgaps $E_{g}$. Depending upon x, the alloys can be either optically transparent or absorbing at 1.3 μm and/or 1.55 μm wavelengths. The concept of preparing direct bandgap group IV carbides XC (X ≡ Si, Ge, Sn) and their polymorphs [i.e., 3C (cubic or zinc-blende (zb)), 2H, 4H, 6H (hexagonal), 9R, 15R (rhombohedral)] on Si substrates has recently offered a paradigm shift in Si photonics concerning monolithic implementation of light emitters. These novel materials [95,96,97,98,99,100,101,102,103,104,105,106,107,108,109,110,111,112,113,114,115,116,117,118,119,120] have demonstrated several incredible properties different from the II-VI, III-V, and III-N compound semiconductors, which make them especially relevant for further investigations.

The growth of crystalline quality zb XC/Si (001) epifilms is a major challenge. The pulsed supersonic free jets technique [121,122,123] was employed earlier for inverse heteroepitaxial growth of Si on SiC to achieve good quality multilayer structures. In MBE, a novel arc plasma C gun source was used to prepare MQWs and SLs [124,125,126]. Ultrahigh UH-CVD, reduced pressure RP-CVD, vertical reactor (V-CVD) [127,128,129,130,131,132,133], and MOCVD techniques have also been employed for achieving different Si_1−x_Ge_x_C/Si, Ge_1−x_Sn_x_C/Si, GeC/SiC, and GeC/Si epilayers [134,135]. Several optical and structural studies have been reported exploiting IR, RSS [136], high-resolution X-ray diffraction (HR-XRD) [137], photoluminescence (PL) [138,139,140], high-resolution transmission electron microscopy (HRTEM), and spectroscopic ellipsometry (SE) techniques [141,142]. The results of electronic, phonon, and structural traits have been found, however, to be drastically different. Information on the lattice dynamics of perfect and/or imperfect solids can be obtained using optical spectroscopy [143,144]. The most efficient approaches considered in assessing the complete phonon dispersions of group IV elemental, III-V, and II-VI compound semiconductors have employed the inelastic neutron scattering (INS) [143,144] and/or inelastic X-ray scattering (IXS) [145] methods. Except for 3C-SiC [145], there exist no phonon measurements for the zb GeC and SnC materials using IXS and/or RSS [136]. Again, INS cannot be exploited to comprehend the vibrational properties of zb XC/Si (001) epifilms because the samples are too lean to obtain measurable signals to resolve modes and branches of phonon dispersions lying very close in frequency. 

Besides their electronic characteristics [103,104,105,106,107], it is necessary to have a systematic assessment of phonons to comprehend the operations of C-based nanostructured (NS) devices. One reason for this requisite is that NS can be used to manipulate thermal transport in solids. This is possible as the dominant heat carriers in semiconductors are phonons having characteristic lengths in the nanometer regime. Examining the dynamical response of NS materials with their impacts on the dielectric environment would provide a major step towards realizing their structural and electronic traits. The other reason is that the assessment of accurate phonon dispersions $\omega_{j}^{SL}\left(\vec{q}\right)$ and density of states (DOS) establishes the basis of fabricating many modern devices [146,147]. Recently, Balandin [148] proposed the concept of “phonon engineering”, which might lead to progress in electronic and opto-electronic devices. As the phonons in SLs differ significantly from the constituent materials, it is anticipated that the acoustic phonon spectra might undergo modifications due to spatial confinement. These changes can result in the emergence of quantized phonon dispersions to cause significant variations in the DOS and hybridization of the lattice modes. Both M-LCM and Rytov’s [149] methodologies are generally unable to produce the complete vibrational spectrum of XC materials. Consequently, their use in predicting the phonon characteristics for LDHs at the nanoscale regime becomes unrealistic.

Like electron waves, the phonon states in SLs can also undergo changes induced by external boundaries [18,19,20]. Despite strong scientific and practical importance, the conclusive experimental evidence of folded acoustic phonons (FAPs), confined optical modes (COMs), as well as interface phonon modes (IPMs) [149,150,151,152] in C-based LDHs is lacking. For the dispersion of light, refractive indices of constituent materials play important roles. To study acoustic modes in SLs, the relevant physical quantities are the elastic constants. As the sound velocity depends weakly on solids, one expects the dynamics of acoustic modes of constituent materials to be quite similar in LDHs. Thus, the acoustic phonons in SLs can be described by an effective sound velocity, which depends on the ratio of layer thicknesses of the comprising materials. Again, FAPs and COMs are widely studied by using RSS, Brillouin scattering, and time-resolved spectroscopy in both lattice-matched and lattice-mismatched superlattice structures [149]. It has been confirmed that if the well and barrier materials show similar spectra, their acoustic phonons would propagate across the superlattice, exhibiting FAPs [150,151,152]. If the optical phonon branches in the wells and barriers are largely separated, then these modes cannot propagate through barriers and wells. Such vibrations are treated as confined, i.e., the phonons will be restricted to each layer, with vibrational amplitudes vanishing in the immediate vicinity of the boundaries of constituent layers. 

From a theoretical standpoint, several calculations of phonon dispersions for bulk XC materials have been performed using the full potential linear augmented plane wave (FP-LAPW), first principles (ab-initio), molecular dynamics, and phenomenological methods [103,104,105,106,107,108,109,110,111,112,113,114,115,116,117,118,119,146]—some of these studies provided atypical results. Except for a preliminary report [153], no methodological studies are available on the phonon dispersions $\omega_{j}^{SL}\left(\vec{q}\right)\;of$ novel strained layer ${\left(XC\right)}_{m}/({YC)}_{n}$ SLs, especially for comprehending the prospects of FAPs, COMs, and IPMs. Due to the large lattice mismatch between SiC–GeC (~5.0%), GeC–SnC (~10.5%), and SiC–SnC (~15.0%) [154], one would expect stress in the planes parallel and perpendicular to the SL interfaces. The optical and electrical processes in GaN-, AlN-, and InN-based MQWs and SLs are remarkably influenced by the existence of graded interfaces [155]. The indium concentration gradient across the GaN/InGaN/AlGaN interfaces was measured by HRTEM, pointing to a typical GaN/InGaN interface width of ~1 nm, while more than twice this value is the thickness of the InGaN/AlGaN interface. Again, in the case of GaN/AlGaN SLs, RSS has provided evidence for the graded alloy interface region to be in the order of 2 nm [156]. In the absence of such data in graded XC/YC SLs, it is interesting to analyze the interfacial thickness’s dependence of their phonon characteristics. 

In this paper, the interest in studying the acoustic and optical phonon traits of C-based SLs is driven by our quest to comprehend the basic properties of NS and their applications in electronic devices, where energy transport plays an important role. The purpose of this work is to use: (a) a classical Rytov model (cf. Section 2.1) [149] to simulate the FAPs; (b) a modified linear-chain model (M-LCM) to study the FAPs, IPMs, and COMs (cf. Section 2.2); and (c) a bond polarizability method for calculating the Raman intensity profiles in the optical phonon frequency region for both the ideal ${\left(XC\right)}_{m}/({YC)}_{n}$ and graded ${\left(XC\right)}_{10-∆}/{\left(X_{0.5}Y_{0.5}C\right)}_{∆}/{\left(YC\right)}_{10-∆}/{\left(X_{0.5}Y_{0.5}C\right)}_{∆}$SLs by meticulously integrating an interfacial layer thickness Δ (≡1–3 monolayers (MLs)) (cf. Section 2.3). Our M-LCM calculations near the zone center ${(q}_{SL}\sim \;0)$ of the mini-Brillouin zone (m-BZ) of the SLs for FAPs are shown to be negligibly affected by varying Δ values except for the relative intensity changes noticed disappearing at higher orders of folding for the wider interfaces. In graded SLs, however, the impact of increasing Δ values on the COMs is quite significant, revealing a large “downward” (“upward”) shift of higher (lower) frequency optical modes [155]. Obviously, these shifts triggered overlapping of confined modes with the neighboring optical phonon branches. Accordingly, our calculations of the Raman scattering spectra using the bond polarizability model revealed enhanced peaks in the middle of the optical phonon frequency regions. The enhancement of Raman intensity profiles was caused either by the collapsing or overlapping of neighboring optical phonon modes. This argument was fully supported by our simulations of atomic displacements, which caused the localization of appropriate phonon modes (cf. Section 3). In C-based SLs, the magnitude of estimated shifts of optical phonons from an elasticity model was found to be equally consistent with the strain-induced changes. We strongly feel that the methodologies adopted here can be extended to other technologically important LDHs and will encourage Raman spectroscopists to perform similar measurements to check our theoretical conjectures.

## 2. Theoretical Background

In semiconductor materials, lattice dynamics play crucial roles in assessing their basic characteristics, including acoustic, thermal, electronic, and optical properties. The benefits of understanding acoustic phonon features are driven by their importance in nanostructured electronics, where energy transport offers tremendous aids via heat removal from state-of-the art integrated circuits to increase efficiency of thermoelectric energy conversion in various NS devices [148]. Artificial translational symmetry in SLs is responsible for the folding of acoustic phonon modes into a smaller m-BZ. Such folding gives rise to additional optical phonon-like features with non-zero frequencies at the zone center of m-BZ (i.e., $q_{SL}\sim 0$). The coupling of such FAPs to light has inspired many Raman spectroscopists [151] to use spontaneous RSS for detecting them. 

### 2.1. Macroscopic Theory of Folded Acoustic Phonons in Superlattices

**Figure 1:**
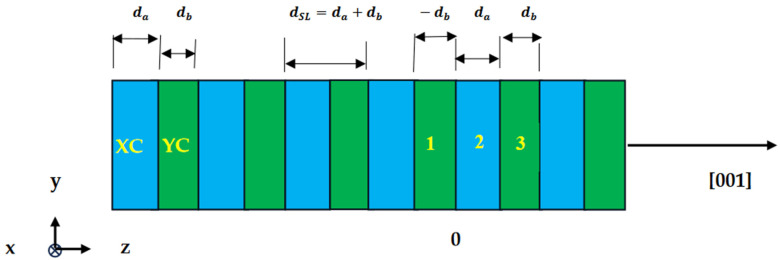
Schematic representation of a (XC)_m_/(YC)_n_ superlattice having two constituent XC/YC materials arranged alternately along the z or [001] direction. The term m represents the number of XC monolayers of thickness $d_{a}$ embedded in between two n number of YC monolayers of thickness $d_{b}$, causing the period $d_{SL}(\equiv d_{a}+d_{b}$) of SL.

Phonons in SLs have been extensively studied using Raman scattering spectroscopy [151]. This experimental method allows for the propagation effects needed to understand the physics of NS with the prospect of using coherent phonons for imaging. Acoustic modes in artificial SLs are modified by periodically compiling arrays of two materials, *a* and *b*, in the *z*-direction. As the sound velocity weakly depends on solids, one expects the dynamics of acoustic modes in constituent materials of layered structures to be similar. Thus, an effective sound velocity may be used to study FAPs by linking them to the ratio of their layer thicknesses $d_{a}$ and $d_{b}$. Since the period of an SL is increased from $a_{o}$ to $d_{SL}$ along the *z*-direction (cf. Figure 1), one would expect a reduction in the BZ from $\frac{2\pi }{a_{o}}$ to $\frac{\pi }{d_{SL}}$, prompting the folding of acoustic modes in the reduced m-BZ [149].

#### Folded Acoustic Phonons

The distribution of FAPs, including the splitting at zone center ($q_{SL}\sim 0$) and at the edge ($q_{SL}\sim \frac{\pi }{d_{SL}}$) of the m-BZ, can be calculated assuming a linear dispersion of the constituent materials:

(1a) ω=qv,

(1b) $$\mathrm{with}\;\;\;\;\;\;\;\;\;\;\;\;\;\;\;\;\;\;\;\;\;\;\;\;\;\;\;\;\;\;\;\;\;\;\;\;\;\;\;\;\;\;\;\;\;\;\;\;\;\;\;\;\;\;\;\;v=\sqrt{\frac{C_{11}}{\rho }}.\;\;\;\;\;\;\;\;\;\;\;\;\;\;\;\;\;\;\;\;\;\;\;\;\;\;\;\;\;\;\;\;\;\;\;\;\;\;\;\;\;\;\;\;\;\;\;\;\;\;\;\;\;\;\;\;$$

Here, v, ρ, and $C_{11}$ are the sound velocity, mass density, and stiffness constant of longitudinal modes along the growth (or *z*-) direction, respectively. In an artificial structure, the two materials *i* (≡*a*, *b*) are characterized by their acoustic impedances $Z_{i}(\equiv v_{i}\times \rho_{i}).$ The equation of one-dimensional elastic waves propagating along the *z*- direction is described as [149]:

(2) $$\frac{\partial }{\partial t}\left(\rho_{a,b}(z)\frac{\partial u_{a,b}(z,t)}{\partial t}\right)=\frac{\partial }{\partial z}\left(c_{11}^{a,b}(z)\frac{\partial u_{a,b}(z,t)}{\partial z}\right)\;,$$

where $u_{a,b}(z,t)$ relates to the displacement. Assuming the material parameters in each layer to be homogeneous, Equation (2) becomes:

(3) $$\rho_{j}\frac{\partial^{2}u_{a,b}(z,t)}{\partial t^{2}}=c_{j}\frac{\partial^{2}u_{a,b}(z,t)}{\partial z^{2}},$$

where the subscript *j* indicates the material properties in the *j^th^* layer. As the system has a translational invariant in the *x* and *y* directions, one can consider harmonic solutions assuming a plane wave propagating along the *z* direction:

(4) $$u\left(z,t\right)=u\left(z\right)\times e^{i\omega t}.$$

Inside each layer *j*, the spatial part *u*(*z*) is articulated in terms of the two counter-propagating plane waves:

(5) $$u_{j}\left(z\right)=A_{j}^{+}e^{iq_{j}z}+A_{j}^{-}e^{-iq_{j}z},$$

where $q_{j}$ is the local wavevector of the plane wave in layer *j*. One may use the boundary conditions at different interfacial layers between 1, 2, and 3:

(6a) $$u_{j}\left(b_{j}\right)=u_{j+1}\left(b_{j}\right),$$

including the continuity of stress and displacement between two consecutive layers, i.e., [149]:

(6b) $$C_{j}{\left.\frac{\partial u_{j}}{\partial z}\right|}_{b_{j}}=C_{j+1}{\left.\frac{\partial u_{j+1}}{\partial z}\right|}_{b_{j}},$$

where the term *b_j_* in Equations (6a) and (6b) indicates the interface between layers *j* and *j* + 1.

With these conditions, Equation (5) for layers 1 and 2 (cf. Figure 1) will take the form:

(7a) $$u_{1}\left(z\right)=A_{1}^{+}e^{iq_{1}z}+A_{1}^{-}e^{-iq_{1}z},$$

and

(7b) $$u_{2}\left(z\right)=A_{2}^{+}e^{iq_{2}z}+A_{2}^{-}e^{-iq_{2}z}.$$

As the system is periodic, one may apply the Bloch theorem for layer 3. The mechanical solution can be put in the form:

(7c) $$u\left(z\right)=\varphi \left(z\right)\times e^{iqz},$$

where φ(z) is a part of the Bloch wave having the same periodicity as that of the SL, $d_{SL}$. Thus, the mechanical wave in layer 3 can be written as:

(7d) $$u_{3}\left(z\right)=e^{iq_{SL}d_{SL}}\times (A_{1}^{+}e^{iq_{1}(z-d_{SL})}+A_{1}^{-}e^{-iq_{1}(z-d_{SL})}).$$

By applying the boundary conditions [cf. Equations (6a) and (6b)] at the interfaces between layers 1 and 2 and between layers 2 and 3, Equations (7a)–(7d) will become:

(8a) $$A_{1}^{+}+A_{1}^{-}=A_{2}^{+}+A_{2}^{-},$$

(8b) $$C_{1}q_{1}\left(A_{1}^{+}-A_{1}^{-}\right)=C_{2}q_{2}{(A}_{2}^{+}-A_{2}^{-}),$$

(8c) $$A_{2}^{+}e^{iq_{2}d_{a}}+A_{2}^{-}e^{-iq_{2}d_{a}}=e^{iq_{SL}d_{SL}}(A_{1}^{+}e^{{-iq}_{1}d_{b}}+A_{1}^{-}e^{iq_{1}d_{b}}),$$

(8d) $${C_{2}q_{2}{(A}_{2}^{+}e^{iq_{2}d_{a}}-A_{2}^{-}e^{-iq_{2}d_{a}})=C}_{1}q_{1}e^{iq_{SL}d_{SL}}\left(A_{1}^{+}e^{{-iq}_{1}d_{b}}-A_{1}^{-}e^{iq_{1}d_{b}}\right),$$

where $q_{j}v_{j}=\omega$ and $v_{j}=\sqrt{\frac{C_{j}}{\rho_{j}}}$ is the speed of sound in layer *j*. From Equations (8a)–(8d), it is straightforward to obtain a homogenous linear SL system with four unknowns. The non-trivial solution of its determinant will provide the dispersion relation between ω and $q_{SL}$:

(9a) $$\cos\left(q_{SL}d_{SL}\right)=\cos\left(\omega \left(\frac{d_{a\;}}{v_{a}}+\frac{d_{b\;}}{v_{b}}\right)\right)-\frac{\varepsilon^{2}}{2}\sin\left(\omega \frac{d_{a\;}}{v_{a}}\right)\sin\left(\omega \frac{d_{b\;}}{v_{b}}\right)\;\;,$$

(9b) $$=\cos\left(\omega \;t_{SL}\right)-\frac{\varepsilon^{2}}{2}\sin\left(\omega \frac{d_{a\;}}{v_{a}}\right)\sin\left(\omega \frac{d_{b\;}}{v_{b}}\right).$$

The above relation, also known as the Rytov equation [149], has the same form as the dispersion relation for electrons in a periodic potential in the Krönig–Penney model. In Equation (9b), $t_{SL}\left(=\frac{d_{SL}}{v_{SL}}\right)$ is the transit time through one period of the SL with $v_{SL}\;[\equiv \frac{d_{SL}}{\left(\frac{d_{a\;}}{v_{a}}+\frac{d_{a\;}}{v_{a}}\right)}]$ as an average sound velocity. The term $\varepsilon \left(\equiv \frac{{|v}_{a}\rho_{a}-v_{b}\rho_{b}|}{{\left({v_{a}\rho_{a}v}_{b}\rho_{b}\right)}^{1/2}}\right)$ in Equations (9a) and (9b) represents a normalized relative difference between acoustic impedances $Z_{i}(\equiv v_{i}\rho_{i}$) of the two bulk constituents. Again, the first term in the right-hand side of Equation (9a) or (9b) describes the folding of an “average” dispersion curve of an SL and thus reflects the geometry of its structure. The second term describes the acoustical modulation, which leads to frequency splitting of the modes both at the center ($q_{SL}\sim 0$) and at the edge ($q_{SL}\sim \frac{\pi }{d_{SL}}$) of the m-BZ. The latter effect is, however, rather small due to the comparable values of acoustical impedances for the typical semiconductor materials, thus $\frac{\varepsilon^{2}}{2}\;\approx 10^{-2}$.

It has been reported by Santos et al. [157] that the gaps in FAPs at the zone center and at the zone edge correspond to eigen displacements with equal amplitudes of both the forward and backward propagating waves. This implies that they do not transport energy. By solving the equation of motion [Equation (2)], the ratio of amplitudes within a layer can be expressed as:

(10) $$\frac{A_{j}^{-}}{A_{j}^{+}}=\pm \frac{2}{\varepsilon^{\prime }}\frac{sin\left[\frac{{\omega (t}_{b}+t_{a})-q_{SL\;}d_{SL}}{2}\right]}{sin\left[\frac{{\omega (t}_{b}-t_{a})-q_{SL\;}d_{SL}}{2}\right]}\;\;\;\mathrm{with}\;\varepsilon^{\prime }\left(=\frac{{(v}_{a}\rho_{a}-v_{b}\rho_{b})}{{(v}_{a}\rho_{a}+v_{b}\rho_{b})/2}\right)$$

where the “+” and “−” signs at the right-hand side of Equation (10) are for layer *b* and layer *a*, respectively, and the term $t_{j}\left(=\frac{d_{j}}{v_{j}}\right)$ is the phase traversal time through layer *j*. The gaps at the zone center are bounded by the frequencies corresponding to the solution of Equation (10) for $q_{SL}\sim \;0$ and $A_{j}^{+}$ = $A_{j}^{-}$, which yields:

(11) $$sin\left[\frac{{\omega (t}_{b}+t_{a})}{2}\right]=\pm \frac{\varepsilon^{\prime }}{2}\;sin\left[\frac{{\omega (t}_{b}-t_{a})}{2}\right],$$

Obviously, the gaps disappear in the case of two equal acoustical impedances or phase traversal times. Thus, the m^th^ zone center frequencies degenerate to

(12) $$\Omega_{m}\approx \frac{2m\pi v_{SL}}{d_{SL}},\;\;\;\;\;\;\;\;\;\;\;\;\;\;\mathrm{with}\;m=0,\;\pm 1,\;\pm 2\ldots ..$$

where m describes the order of FAPs for the crossings at the center (edge) of the m-BZ. If the gap is small compared to $\Omega_{m}$, as is usually the case, one can assume the solution of Equation (11) to be located symmetrically around $\Omega_{m}$, i.e., $\Omega_{\pm m}=\Omega_{m}\pm {∆\Omega }_{m}/2$. Since the right-hand side of Equation (11) is a slowly varying function of ω, it can be replaced by $\Omega_{m}$. After expanding the left-hand side of Equation (11) around $\Omega_{m}$, one obtains:

(13) $$\frac{{∆\Omega }_{m}}{2}\;\approx \frac{v_{SL}}{d_{SL}}\left|\varepsilon^{\prime }sin\left(m\pi \frac{(1-\alpha )v_{b}-\alpha v_{a}}{(1-\alpha )v_{b}+\alpha v_{a}}\right)\right|,$$

where $\alpha =\frac{d_{b}}{d_{SL}}$. It is clearly noticed that the magnitude of the gap ${∆\Omega }_{m}$ is comparable for all m values on the assumption of a linear dispersion. It displays an oscillatory behavior as a function of α and is proportional to the modulation parameter $\varepsilon^{\prime }$ and to the averaged velocity $v_{SL}$ and inversely proportional to the period $d_{SL}$. Note that all the zone center gaps vanish for the same value of $\alpha_{c}=\frac{v_{b}}{v_{a}+v_{b}}.$

Similarly, the gaps at the zone boundary are calculated by setting $q_{SL}=\pi /d_{SL}$ in Equation (10). This will give rise to:

(14) $$\Omega_{m}^{b}=|2m+1|\pi \frac{v_{SL}}{d_{SL}}\;\mathrm{with}\;m=0,\;\pm 1,\;\pm 2,\;\ldots \;\;\;\;\;\;\;\;\;\mathrm{and}$$

(15) $$\frac{∆{\;\Omega }_{m}^{b}}{2}\approx \frac{v_{SL}}{d_{SL}}\left|\varepsilon^{\prime }cos\left(\frac{\left|2m+1\right|}{2}\pi \frac{(1-\alpha )v_{b}-\alpha v_{a}}{(1-\alpha )v_{b}+\alpha v_{a}}\right)\right|.$$

Once again, the choice of individual layer thicknesses in SLs determines (cf. Section 3) the absolute value of acoustic minigap frequencies and their span. Obviously, by decreasing the period $d_{SL},$ one can increase the span and the central frequency of the minigaps. Choosing material combinations with a large ε value also allows for widening the forbidden frequency intervals.

### 2.2. Macroscopic Theory of Confined Optical Phonons in Superlattices 

In polar materials, the optical phonons offer major contributions to develop the nano-photonic devices in the MIR—FIR region, as they enable excitation of phonon polaritons to extend the lower losses and higher quality factors compared to the plasmon counterparts. Despite their potential use in optoelectronic applications, no efforts have been made to comprehend the optical phonon traits in C-based LDHs. In epitaxially grown SLs, one expects a large mismatch between the lattice constants of constituent XC/YC materials causing the steep strain gradients and atypical changes in the interfacial chemistry. These factors are expected to instigate significant variations in the electronic and phonon characteristics affecting their optical properties.

#### Confined Optical Phonons: Strain Effects 

Unlike FAPs, one expects strain-induced shifts in optical phonons in highly lattice-mismatched C-based (XC)_m_/(YC)_n_ SLs. Comprehending changes in optical phonons in LDHs is important not only from an application viewpoint for designing long-wavelength optoelectronic devices but also from a basic research perspective. The impacts of strain on the shifts of longitudinal optical ($\omega_{LO}$) modes can be estimated by RSS in the backscattering configuration using incident light perpendicular to the interface plane and evaluating changes in $∆\omega_{LO}$ from the bulk values. 

In this configuration, one cannot estimate the variations in $∆\omega_{TO}$ phonons induced by directional stress. Biaxial stress can cause the splitting of LO-TO phonons. For identifying the variations in singlet ${∆\omega }_{LO}$ and doublet ${∆\omega }_{TO}$ modes in the layered structures, it is required to use Raman scattering spectroscopy with incident light parallel and perpendicular to the interface planes. In the absence of such experimental data in strained layer (XC)_m_/(YC)_n_ SLs, the values of $∆\omega_{LO}$ and ${∆\omega }_{TO}$ can be calculated using an elasticity theory [150,151]. 

One must note that the epitaxially grown XC/YC LDHs are strongly influenced by the large (5 to 15%) disparities in the lattice constants (*a*_i_ and *a*_j_) of their constituent binary materials. In terms of *a*_i_ and *a*_j_, the in-plane lattice parameter $a_{\left|\right|}$ to the interface can be estimated [150,151]:

(16a) $$a_{||\;}=a_{i}\left(1-\frac{f}{1+(\frac{G_{i}d_{i}}{G_{j}d_{j}})}\right)=\left(\frac{a_{i}G_{i}d_{i}+a_{j}G_{j}d_{j}}{G_{i}d_{i}+G_{j}d_{j}}\right),$$

where $d_{i}$ and $d_{j}$ are the layer thicknesses, *f* is the lattice mismatch between unstrained bulk lattice constants $f=(a_{i}-a_{j})/a_{i}$, and $G_{i}$ is the shear modulus:

(16b) $$G_{i}=2(C_{11}^{i}+C_{12}^{i}-2(C_{12}^{i}{)}^{2}/C_{11}^{i}).$$

For zb XC compounds, the values of elastic constants are listed in Table 1. Components of the strain for in-plane $\varepsilon_{\left|\right|}^{i}$ and perpendicular $\varepsilon_{⊥}^{i}$ to the interface of the SLs are calculated:

(17a) $$\varepsilon_{xx}^{i}=\varepsilon_{yy}^{i}=\varepsilon_{\left|\right|}^{i}=\left({(a}_{\left|\right|}-a_{i})/a_{i}\right),$$

(17b) $$\varepsilon_{⊥}^{i}=-2\;(\frac{C_{12}^{i}}{C_{11}^{i}}){\;\varepsilon }_{\left|\right|}^{i}.$$

Strain-induced frequency shifts of ${∆\omega }_{LO}$ and ${∆\omega }_{TO}$ modes are obtained by using the expressions [150]:

(18a) $$∆\omega_{LO}^{i}=\frac{\left[p^{i}S_{12}^{i}+q^{i}(S_{11}^{i}+S_{12}^{i})\right]}{S_{11}^{i}+S_{12}^{i}}\frac{\varepsilon_{\left|\right|}^{i}}{\omega_{LO}^{i}},$$

(18b) $$∆\omega_{TO}^{i}=\frac{\left[p^{i}{(S_{11}^{i}+S}_{12}^{i})+q^{i}(S_{11}^{i}+{3S}_{12}^{i})\right]}{S_{11}^{i}+S_{12}^{i}}\frac{\varepsilon_{\left|\right|}^{i}}{2\;\omega_{TO}^{i}},$$

where the terms $S_{11}^{i}$ and $S_{12}^{i}$ are the elastic compliance constants (see: Table 1) and $p^{i}$ and $q^{i}$ are the phonon deformation potential parameters proportional to the changes in the spring constants induced by strain. 

**Table 1:** Elastic constants C_ij_ (10^11^ dyn/cm^2^), compliance constant S_ij_ (10^−12^ cm^2^/dyn), *a*_o_ lattice constant (Ẳ), and phonon frequencies at (Γ, X, L) critical points (cm^−1^) for 3C-SiC and zb GeC used for calculating the necessary interatomic force constants for the rigid-ion model and modified linear-chain model for simulating the phonon dispersions of bulk and (SiC)_m_/(GeC)_n_ superlattices.

Parameters	Exptl. ^a^	Exptl. ^b^	Calc. ^c^	Calc. ^c,d^	Calc. ^c,d^
	SiC			GeC	SnC
C_11_	39.0	35.2	39.0	35.8	24.6
C_12_	14.2	14.0	14.3	12.2	11.3
C_44_	25.6	23.3	25.2	21.4	14.3
S_11_			0.318	0.338	0.572
S_12_			−0.0850	−0.0859	−0.180
S_44_			0.391	0.467	0.699
*a*_o_	4.3596	4.32	4.36	4.59	5.13
*v*			1.1013 × 10^6^	7.8454 × 10^5^	6.179 × 10^5^
*ρ*			3.2157	5.8164	6.4318
LO(Γ)	972	974	974	749	558
TO(Γ)	796	793	797	626	456
LO(X)	829	830	828	697	512
TO(X)	761	759	760	617	454
LA(X)	640	644	639	348	216
TA(X)	373	373	373	211	141
LO(L)	838	850	857	705	524
TO(L)	766	770	787	621	454
LA(L)	610	605	591	326	214
TA(L)	266	260	250	166	102

^a^ Ref. [145], ^b^ Ref. [137], ^c^ Ref. [154], ^d^ Refs. [139,142].

In Equation (18a), $∆\omega_{LO}^{i}$ indicates the shift in singlet-type mode vibrating parallel to the <001> axis, while $∆\omega_{TO}^{i}$ in Equation (18b) suggests the shift of doublet-type mode vibrating perpendicular to the <001> axis. This splitting occurs due to biaxial stress, which makes the structure quasi two dimensional. With the parameter values listed in Table 1 and using Equations (18a)–(18b), we estimated the values and established |$∆\omega_{LO}^{i}$| > |$∆\omega_{TO}^{i}$| for the SiC/GeC, GeC/SnC, and SiC/SnC SLs (cf. Section 3). To comprehend the significance of simulated shifts in the optical phonons, the calculations of Raman intensity profiles in the SLs were also performed based on the M-LCM approach by using a bond-polarizability method (cf. Section 3.1.5).

### 2.3. Modified Linear-Chain Model for Superlattices 

Calculations of phonon dispersions $\omega_{j}\left(\vec{q}\right)$ for the bulk zb XC, YC materials are available [146,154] along the high-symmetry directions in the BZ. With the appropriate bulk phonon frequencies, the necessary force constants of the M-LCM method are obtained to simulate $\omega_{j}^{SL}(\vec{q})$ for ideal (XC)_m_/(YC)_n_ SLs, considering m = n = 10. In this calculation, the plane of atoms in the actual SL is represented by an atom in the linear chain. This picture allows the associated phonons propagating along the growth (*z*-) direction to be described by one-dimensional sets of equations of motion. The $\omega_{j}^{SL}(\vec{q})$ for the graded ${\left(XC\right)}_{10-∆}/{\left(X_{0.5}Y_{0.5}C\right)}_{∆}/{\left(YC\right)}_{10-∆}/{\left(X_{0.5}Y_{0.5}C\right)}_{∆}$ SLs, are carefully simulated by meticulously integrating the interfacial layer thickness (cf. Section 3.1.1, Section 3.1.2, Section 3.1.3, Section 3.1.4, Section 3.1.5 and Section 3.1.6) Δ and varying its value from 1 to 3 MLs. 

By incorporating the phonon dispersions $\omega_{j}\left(\vec{q}\right)$ of bulk materials, the thermal conductivity *κ* can be calculated by using [47]:

(19) $$\kappa_{j}=\sum_{i}C_{ph}\left(\omega_{i}\right){\left(\frac{\partial \omega }{\partial q_{i}}\right)}^{2}\tau \;.$$

In Equation (19), the term $\omega_{i}$ represents an i^th^ phonon mode, *C*_ph_ is the phonon specific heat, $v_{gi}$ $\left(\equiv \frac{\partial \omega }{\partial q_{i}}\right)$ is the group velocity, and τ represents the relaxation time. It should be noted that the major limitation for determining *κ* has been the mean free path (MFP) $\Lambda \left(\equiv \frac{v_{g}}{\tau }\right)$, as τ is controlled by the phonon–phonon scattering $\tau_{pp}$, impurity scattering $\tau_{imp}$, and boundary scattering $\tau_{B}$. In SLs, due to dimensionality confinement, the term $\tau_{B}$ is pronounced, which can severely modify heat transport characteristics [47]. The possibility of tuning thermal conductivity in LDHs via phonon engineering has been of extreme importance, as it can lead to numerous breakthroughs, including a high figure of merit, improved energy efficiency, etc. It has been shown theoretically [148] that confinement-induced modification of the acoustic phonon spectrum in free space ultrathin films and nanowires leads to a significant decrease in the in-plane *κ*. Obviously, these facts suggest that the study of $\omega_{j}^{SL}(\vec{q})$ is quite cumbersome and requires realistic lattice dynamical methods.

#### 2.3.1. Raman Scattering

For understanding the structural characteristics of different types of short period SLs, the measurement of phonons by Raman scattering spectroscopy has played a valuable role. In zb materials, the selection rules forbid $\omega_{TO}$ modes in the backscattering geometry of the (001) face. Symmetry arguments suggest, however, that only the $\omega_{LO}$ modes of perfect SLs can be observed. In the absence of such data for (XC)_m_/(YC)_n_ SLs, we performed simulations of Raman intensity profiles using an M-LCM method in the framework of a bond polarizability model [25]. In the graded SLs, we considered alloyed interfaces in the virtual crystal approximation, where the interface structure was assumed to have both constituents with equal proportion. Interfacial layer thickness Δ (≡1 to 3 MLs) was methodically included in the M-LCM model for simulating $\omega_{j}^{SL}(\vec{q})$. 

#### 2.3.2. Raman Intensity Profiles in Superlattices

Following Zhu and Chao [25] and adopting a bond polarizability model, we calculated the Raman intensity profiles $I\left(\omega \right)$ (cf. Section 3.1.5 and Section 3.1.6) and atomic displacements $u_{j}^{SL}\left(\vec{q}\right)$ (cf. Section 3.1.7) for both ideal (XC)_m_/(YC)_n_ and graded ${\left(XC\right)}_{10-∆}/{\left(X_{0.5}Y_{0.5}C\right)}_{∆}/{\left(YC\right)}_{10-∆}/{\left(X_{0.5}Y_{0.5}C\right)}_{∆}$ SLs. Raman intensity calculations were performed using:

(20a) $$I_{\mathrm{xx}}\;(\omega )\;\propto {\left|\sum_{A}\alpha_{xx,A}(u_{1z}-u_{3z})\right|}^{2},$$

for modes of A_1_ type symmetry and

(20b) $$I_{\mathrm{xy}}\;(\omega )\;\propto {\left|\sum_{A}\alpha_{xy,A}(u_{1z}+u_{3z}-2u_{0z})\right|}^{2},$$

for modes having B_2_ symmetry. To calculate $I\left(\omega \right)$ using Equation (20a), we assumed fixed values of polarizability constants $\alpha_{ij,A}$ throughout the SLs. One should note that the summation in Equation (20b) runs over all A atoms represented either by X or Y atoms, with u_0_ being the displacements for each of these, while u_1_ and u_3_ represent the displacements of their nearest-neighbor C atoms.

## 3. Numerical Computations, Results, and Discussions

Raman scattering spectroscopy is frequently used for understanding the phonon characteristics of both the lattice-matched and lattice-mismatched LDHs. It has been substantiated that if wells and barriers show similar vibrational spectra, the propagation of acoustic modes exhibit zone folding. On the other hand, the optical modes in wells (barriers) cannot propagate through barriers (wells) if their phonon branches are well separated. Thus, the optical modes in wells will be confined, exhibiting properties independent of the barriers. In (XC)_m_/(YC)_n_ SLs, we reported our systematic simulations of the folded longitudinal acoustic phonons, confined optical modes, and interface modes. Calculations of the FAPs were performed using a classical Rytov model (cf. Section 3.1.1), while the lattice dynamics and Raman intensities were achieved by M-LCM in the framework of a bond-polarizability scheme. Theoretical results are compared/contrasted and discussed (cf. Section 3.1.2, Section 3.1.3, Section 3.1.4, Section 3.1.5 and Section 3.1.6) with the RSS data of other SLs.

### 3.1. Phonons in Superlattices

Due to strong covalency of C-based materials, Raman scattering has high efficiency for extracting valuable information about strain, which can have substantial impacts on their vibrational and electronic properties. As the lattice vibrations in SLs depend on the bonds connecting different types of atoms in the constituent materials and interfacial layers, the RSS can provide unique fingerprints for identifying the nature of their chemical structures [150,155,156,157,158]. Earlier, in III-nitride-based MQWs and SLs, the Raman scattering spectroscopy validated the role of interfacial structures by significantly modifying their optical and electrical characteristics [155]. In the absence of RSS data, it is interesting to analyze the interfacial thickness Δ (≡0–3 MLs) dependence of optical phonons in the graded ${\left(XC\right)}_{10-∆}/{\left(X_{0.5}Y_{0.5}C\right)}_{∆}/{\left(YC\right)}_{10-∆}/{\left(X_{0.5}Y_{0.5}C\right)}_{∆}$ SLs (cf. Section 3.1.1, Section 3.1.2, Section 3.1.3, Section 3.1.4, Section 3.1.5 and Section 3.1.6). 

#### 3.1.1. Rytov’s Model for Folded Acoustic Phonons 

**Figure 2:**
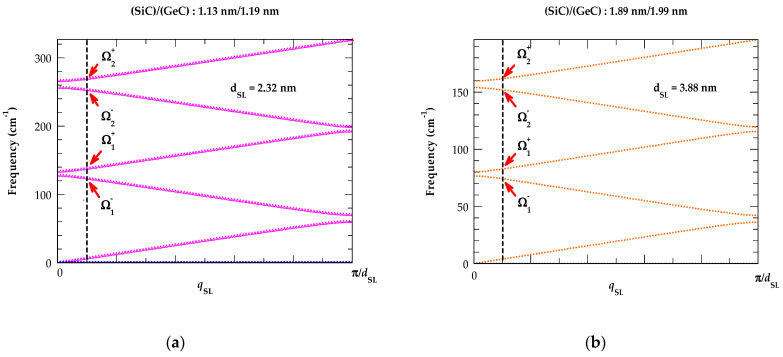

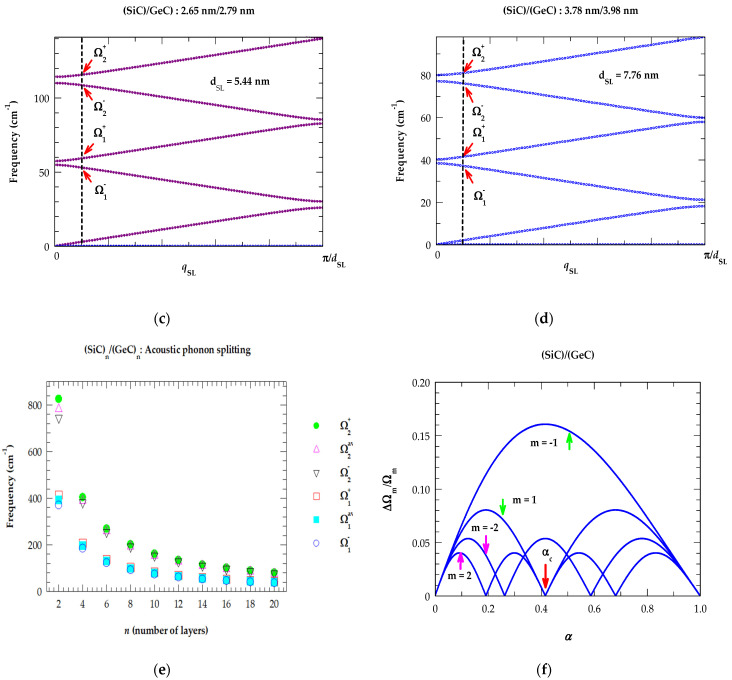
Results of the calculated folded longitudinal acoustic phonons based on Rytov’s model for (SiC)_m_/(GeC)_n_ superlattices using m = n: (**a**) 6/6, (**b**) 10/10, (**c**) 14/14, (**d**) 20/20; (**e**) variation of first- and second-order folded acoustic phonon splitting (see: Table 2) and their average values (shown by symbols on the right-hand side of (**e**)) are plotted as a function of the number of monolayers (m = n) for (SiC)_m_/(GeC)_n_ superlattices from 2 to 20; (**f**) variation of the acoustical gaps at the lower zone center (m > 0) and zone edge (m < 0) normalized to the corresponding average frequency as a function of the relative thickness for SiC/GeC superlattice (see: text).

Rytov’s elastic continuum model (cf. Section 2.1) was employed for simulating the folded acoustic phonon dispersions of (SiC)_m_/(GeC)_n_, (GeC)_m_/(SnC)_n_, and (SiC)_m_/(SnC)_n_ SLs. Here, we chose an equal number of layers for each constituent m (=n) and varied the SL periods $d_{SL}\;\left(\equiv d_{a}+d_{b}\right).$ The simulations (see: Figure 2a–d) were performed for different (SiC)_m_/(GeC)_n_ [SiC)_m_/(SnC)_n_ and (GeC)_m_/(SnC)_n_] SLs by considering $d_{SL}$ values ranging from 0.776 nm to 7.76 nm [0.818 nm to 8.18 nm and 0.838 nm to 8.38 nm]. As an example, we display in Figure 2a–d results of (SiC)_m_/(GeC)_n_ for four different $\;d_{SL}(\equiv$ 2.32 nm, 3.88 nm, 5.44 nm, and 7.76 nm) using magenta, orange, violet, blue color points, respectively.

Black vertical lines are drawn near the zone center to identify the calculated first- $(\Omega_{1}^{-},$ $\Omega_{1}^{+})$ and second order $(\Omega_{2}^{-},$ $\Omega_{2}^{+})$ phonon splitting of the FAPs in each structure. The perusal of Figure 2a–d clearly reveals that as the SL period $d_{SL}$ increased, the values of first- and second-order longitudinal acoustic phonon frequencies not only became consistently lower, but the separation between $\Omega_{1}^{-}-\Omega_{1}^{+}$ and $\Omega_{2}^{-}-\Omega_{2}^{+}$ decreased considerably. Our theoretical predictions reported in Table 2 and Figure 2e are in very good agreement with the existing RSS measurements available for different semiconductor SLs [150,155,156,157,158]. In SiC/GeC system, the calculated values of the magnitudes for acoustic gaps normalized to the corresponding average frequency $\left(\frac{{∆\Omega }_{m}}{\Omega_{m}}\right)$ are plotted in Figure 2f as a function of relative SL thickness α for m = −1, 1, −2, 2. One must note that this ratio does not depend on the SL period $d_{SL}$, and every zone-center gap vanishes at a critical value of $\alpha_{c}$:

(21) $$\alpha_{c}=\frac{v_{b}}{\left(v_{a}+v_{b}\right)}\;,$$

which is ~0.42 for the SiC-GeC system.

**Table 2:** Calculated first- and second order folded acoustic phonon frequency (cm^−1^) splitting $\Omega_{i}^{\pm }$ (i = 1, 2) near the Brillouin zone center by using a classical Rytov model for different periods of (SiC)_m_/(GeC)_n_, (GeC)_m_/(SnC)_n_, and (SiC)_m_/(SnC)_n_ superlattices (see text).

	(SiC)_m_/(GeC,)_n_				(GeC)_m_/(SnC)_n_				(SiC)_m_/(SnC)_n_			
m/n	$\Omega_{1}^{-}$	$\Omega_{1}^{+}$	$\Omega_{2}^{-}$	$\Omega_{2}^{+}$	$\Omega_{1}^{-}$	$\Omega_{1}^{+}$	$\Omega_{2}^{-}$	$\Omega_{2}^{+}$	$\Omega_{1}^{-}$	$\Omega_{1}^{+}$	$\Omega_{2}^{-}$	$\Omega_{2}^{+}$
2/2	299.31	332.5	615.2	648.2	259.4	287.4	532.12	561.41	310.8	321.1	627.0	636.4
4/4	149.7	166.3	307.6	324.1	129.7	143.7	266.1	280.7	149.7	160.6	313.5	318.2
6/6	99.8	110.9	205.1	216.1	86.5	95.8	177.4	187.2	103.6	107.1	209.0	212.2
8/8	74.9	83.2	153.8	162.1	64.9	71.9	133.1	140.4	77.7	80.3	156.8	159.1
10/10	59.9	66.6	123.1	129.7	51.9	57.5	106.5	112.3	62.2	64.3	125.4	127.3
12/12	49.9	55.5	102.6	108.1	43.3	47.9	88.7	93.6	51.8	53.6	104.5	106.1
14/14	42.8	47.6	87.9	92.6	37.1	41.1	76.1	80.2	44.4	45.9	89.6	91.0
16/16	37.5	41.6	76.9	81.1	32.5	36.0	66.6	70.2	38.9	40.2	78.4	79.6
18/18	33.3	37	68.4	72.1	28.9	32.0	59.2	62.4	34.6	35.7	69.7	70.8
20/20	30.0	33.3	61.6	64.9	26.0	28.8	53.3	58.2	31.1	32.2	62.7	63.7

From Figure 2a–d, it is obvious that as the wave vector $q_{SL}$ increases from zero, the separation ${∆\Omega }_{m}$ between the folded acoustic branches increases due to acoustic dispersions. For a smaller value of $q_{SL}$, the splitting ${∆\Omega }_{m}$ of the doublet can be expressed as:

(22) $${∆\Omega }_{m}(q_{SL})={\left({\Omega_{m}(0)}^{2}+4\;v_{SL}^{2}q_{SL}^{2}\right)}^{1/2},$$

where the two contributions for $q_{SL}d_{SL}\;\sim \varepsilon$ on the right-hand side of Equation (22) are of the same order of magnitude. For SiC/GeC SL, the estimated value of ε is ~0.25 and from a typical Raman scattering spectroscopy measurement $q_{SL}\;is\;\sim$10^6^ cm^−1^. Clearly, this leads to $d_{SL}$ ~25.4 Ẳ or 2.54 nm. Thus, in a larger-period SiC/GeC SL, the calculated value of splitting noticed in the doublet of the acoustic modes essentially reflects the dispersions reported in Figure 2a–d. Similar results of the calculated FAPs are perceived for the other (GeC)_m_/(SnC)_n_ and (SiC)_m_/(SnC)_n_ SLs, with a summary of results reported in Table 2. 

#### 3.1.2. Lattice Dynamics

Recently, theoretical simulations of phonon dispersions $\omega_{j}(\vec{q})$ for the zb XC (X = Si, Ge, and Sn) materials have appeared in the literature [146,154]. A closer look confirms significant divergences in their optical and acoustical phonon branches. The variations in phonon characteristics $\omega_{j}(\vec{q})$ are linked to the difference in masses of their common C anion (12.01 amu) and X cation (Si = 28.09 amu; Ge = 72.64 amu, Sn = 118.71 amu) atoms. Unlike the traditional GaAs-AlAs SL, where acoustic phonons of the two constituent materials overlapped due to a common heavier As anion, their optical phonon branches are well separated due to the lower cation Ga and Al masses. However, the situation in XC-YC SLs is quite different. In SiC-GeC (for example), except for a few overlapping acoustic modes between 0–350 cm^−1^, there exist SiC $\omega_{LA}$ phonons in the 350–630 cm^−1^ region with well separated optical modes appearing between 750 and 974 cm^−1^. 

**Figure 3:**
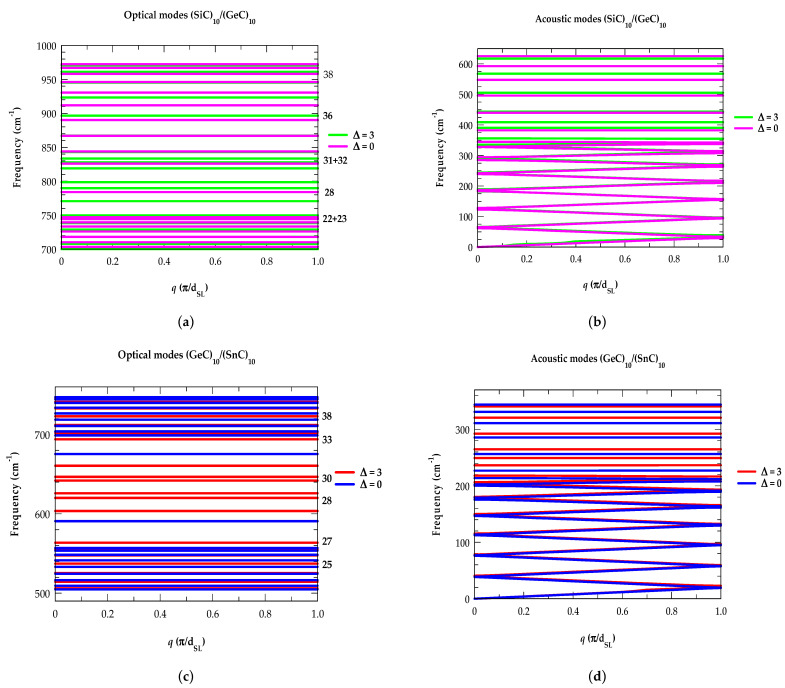

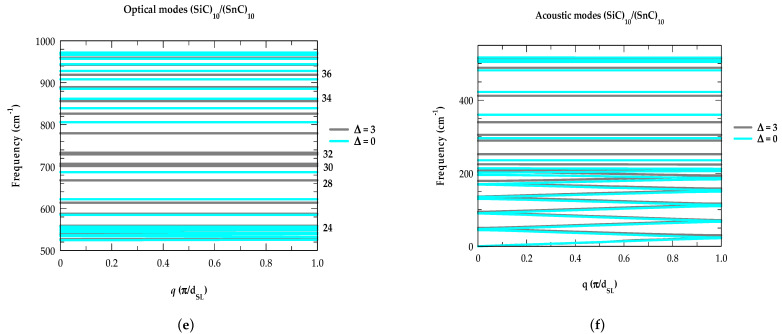
Modified linear-chain model (M-LCM) calculations of phonon dispersions $\omega_{j}^{SL}\left(\vec{q}\right)$ for graded ${\left(SiC\right)}_{10-∆}/{\left({Si}_{0.5}{Ge}_{0.5}C\right)}_{∆}/{\left(GeC\right)}_{10-∆}/{\left({Si}_{0.5}{Ge}_{0.5}C\right)}_{∆}$ SLs with two interfacial layer thicknesses ∆ (≡0 and 3 MLs): (**a**) Confined optical modes (COMs) with specific values listed on the left-hand side are significantly influenced by ∆ (≡3), causing downward (upward) shifts of phonon frequencies; (**b**) folded acoustic modes (FAMs) on the right-hand side are weakly affected by $∆\;\left(\equiv 3\right)$ and corroborated well with Rytov’s model (see: Table 2). Similar calculations for ${\left(GeC\right)}_{10-∆}/{\left({Ge}_{0.5}{Sn}_{0.5}C\right)}_{∆}/{\left(SnC\right)}_{10-∆}/{\left({Ge}_{0.5}{Sn}_{0.5}C\right)}_{∆}$ (**c**,**d**) and ${\left(SiC\right)}_{10-∆}/{\left({Si}_{0.5}{Sn}_{0.5}C\right)}_{∆}/{\left(SnC\right)}_{10-∆}/{\left({Si}_{0.5}{Sn}_{0.5}C\right)}_{∆}$ SLs (**e**,**f**) revealed features identical to those reported in (**a**,**b**) (see text).

In Figure 3a–f, we display our M-LCM results of phonon dispersions $\omega_{j}^{SL}(\vec{q})$ [optical phonons (left-panel) and acoustical modes (right-panel)] for the graded ${\left(XC\right)}_{10-∆}/{\left(X_{0.5}Y_{0.5}C\right)}_{∆}/{\left(YC\right)}_{10-∆}/{\left(X_{0.5}Y_{0.5}C\right)}_{∆}$ SLs using two values of interfacial layer thickness $∆\;\left(\equiv 0,3\right).$ In the ideal (XC)_10_/(YC)_10_ case (∆ ≡ 0), the SL exhibits 20 acoustic and 20 optical phonon branches. In this context, we indicate the 40 vibrational modes by using numbers with increasing frequencies. Modes 1–13 representing FAPs are found to be highly dispersive (see: Figure 3b); the other 7 modes are non-dispersive, as usually found for optical modes. For ${\left(SiC\right)}_{10-∆}/{\left({Si}_{0.5}{Ge}_{0.5}C\right)}_{∆}/{\left(GeC\right)}_{10-∆}/{\left({Si}_{0.5}{Ge}_{0.5}C\right)}_{∆}$ SLs, the perusal of Figure 3a,b reveals some interesting features: (a) in agreement with the existing results of phonon dispersions for binary zb SiC and GeC materials [146,154], our simulations of $\omega_{j}^{SL}(\vec{q})$ in SiC/GeC SLs confirms the FAPs (see: Figure 3b) appearing between 0 to 350 cm^−1^ [the common overlapping acoustic phonon region of SiC–GeC] while the other acoustic modes fall in the frequency range of 350 to 630 cm^−1^ and behave as confined modes in the SiC layer, (b) the calculations of FAPs by M-LCM have corroborated the results derived earlier using Rytov’s model (see: Table 2), (c) the FAPs are weakly affected by varying ∆, and (d) the COMs are significantly influenced by ∆ (≡3) causing downward (upward) shifts of higher (lower) frequency optical modes (cf. Section 3.1.5). Results for other strained layer ${\left(GeC\right)}_{10-∆}/{\left({Ge}_{0.5}{Sn}_{0.5}C\right)}_{∆}/{\left(SnC\right)}_{10-∆}/{\left({Ge}_{0.5}SnC\right)}_{∆}$ (Figure 3c,d) and ${\left(SiC\right)}_{10-∆}/{\left({Si}_{0.5}{Sn}_{0.5}C\right)}_{∆}/{\left(SnC\right)}_{10-∆}/{\left({Si}_{0.5}{Sn}_{0.5}C\right)}_{∆}$ SLs (Figure 3e,f)) have revealed features identical to those reported in Figure 3a,b.

#### 3.1.3. Impact of Strain on the Confined Optical Phonons in Superlattices

The impact of reducing the number of individual layers for studying the role of interfacial thickness ∆ on phonon traits in novel material combinations has been and still is a considerable challenge. If the vibrational modes of different materials in SLs are well separated with frequencies occurring at distinctive locations, the phonons become confined in each constituent layer. Such effects have been extensively studied by Raman scattering and infrared spectroscopies [150,151]. In the long wavelength limit, it has been well established that in SLs the confinement of optical phonon frequencies exhibits significant changes as compared to the phonon energies of individual layers. Besides confinement, the impact of strain-induced shifts and diffusion of atoms for creating interfacial layers on the optical phonon modes has also been observed experimentally [155,156] in different SLs. Since no Raman scattering data are available for highly strained novel (XC)_m_/(YC)_n_ SLs, we report here our theoretical calculations of optical phonon frequency shifts by using elasticity (cf. Section 3.1.4) and M-LCM (cf. Section 3.1.6) methodologies [20,21].

#### 3.1.4. Elasticity Method

Incorporating appropriate data from the literature for bulk 3C-SiC, zb GeC, and SnC materials, we have listed in Table 1 our calculated values of phonons, lattice constants ($a_{o}$), elastic- ($C_{ij}$) and compliance constants (S_ij_), sound velocity (v), mass density (ρ), etc. These quantities helped us evaluate the in-plane ($\varepsilon_{\left|\right|}$) and perpendicular ($\varepsilon_{⊥}$) strains at the interface of each (SiC)_m_/(GeC)_n_, (GeC)_m_/(SnC)_n_, and (SiC)_m_/(SnC)_n_ superlattice system. In the backscattering configuration, we have estimated strain-induced shifts in the optical phonon frequencies for (XC)_m_/(YC)_n_ SLs. Using identical periods $d_{SL}$ of SLs, the calculated results of $∆\omega_{LO},∆\omega_{TO},\varepsilon_{\left|\right|},$ and $\varepsilon_{⊥}$ are reported in Table 3 for SiC-GeC, GeC-SnC, and SiC-SnC systems, respectively. From Table 3, one may note that the bulk SiC LO [TO] phonon frequencies are shifted $∆\omega_{LO}^{SiC}$ [$∆\omega_{TO}^{SiC}$] from −16.1 cm^−1^ to −45 cm^−1^ [−0.565 cm^−1^ to −1.58 cm^−1^] as the number of layers m/n in the SL varied from 8/2 to 8/10, respectively. 

Obviously, for the strained layer (SiC)_m_/(GeC)_n_ SLs these results suggested appropriate downward changes of LO (TO) phonon frequencies from ~958 cm^−1^ to 929 cm^−1^ [796.4 cm^−1^ to 795.4 cm^−1^]. However, the phonon frequencies of zb GeC LO (TO) modes caused upward shifts $∆\omega_{LO}^{GeC}$ [$∆\omega_{TO}^{GeC}$] from 25.2 cm^−1^ to 44.9 cm^−1^ [0.959 cm^−1^ to 1.71 cm^−1^] as the number of m/n layers varied from 8/10 to 8/2 in the strained SLs, respectively. Moving from (SiC)_m_/(GeC)_n_ → (GeC)_m_/(SnC)_n_ → (SiC)_m_/(SnC)_n_ SLs, the calculations have clearly revealed a consistent increase with the downward (upward) changes in the zb GeC [SnC]-like $∆\omega_{LO}^{GeC}$ [$∆\omega_{LO}^{SnC}$] and zb SiC [SnC]-like $∆\omega_{LO}^{SiC}$ [$∆\omega_{LO}^{SnC}$] modes. We strongly believe that these changes in the optical phonon frequencies are associated to the increase in the in-plane $\varepsilon_{\left|\right|}$ and perpendicular $\varepsilon_{⊥}$ strain parameters (see: Table 3). Moreover, the shifts of XC-YC $∆\omega_{LO}$ mode frequencies estimated from the simulated Raman intensity profiles for graded SLs using M-LCM (cf. Section 3.1.6) in the framework of bond-polarizability models provided strong corroboration for the results derived from the elasticity approach. 

#### 3.1.5. Raman Intensity Profiles in Ideal Superlattices 

Besides studying the characteristic phonon features, the Raman scattering spectroscopy allowed for observing additional structures caused by different interfaces in LDHs. In the lack of such data for novel C-based (XC)_m_/(YC)_n_ SLs, it is necessary to simulate Raman intensity profiles. Although the lattice dynamical calculations are known for the bulk SiC, GeC, and SnC materials [146,154], there exist either limited [153] or no reports for comprehending the phonon dispersions $\omega_{j}^{SL}\left(\vec{q}\right)$ of their SLs. To understand the impact of interfacial layer thickness on $\omega_{j}^{SL}\left(\vec{q}\right)$ as well as on Raman spectra, we presented here our systematic results for both the ideal (XC)_m_/(YC)_n_ and graded ${\left(XC\right)}_{10-∆}/{\left(X_{0.5}Y_{0.5}C\right)}_{∆}/{\left(YC\right)}_{10-∆}/{\left(X_{0.5}Y_{0.5}C\right)}_{∆}$ SLs using an M-LCM approach and a bond polarizability methodology [24]. In (SiC)_m_/(GeC)_n_ SLs, while the $\omega_{LA}$ phonon branch of bulk SiC overlaps the $\omega_{TA},\;{\;\omega }_{LA}$ modes and partially covers $\omega_{TO}$ modes of zb GeC, their optical phonons are well separated. It is, therefore, interesting to simulate thickness-dependent Raman intensities of the optical phonons for their SLs. 

**Figure 4:**
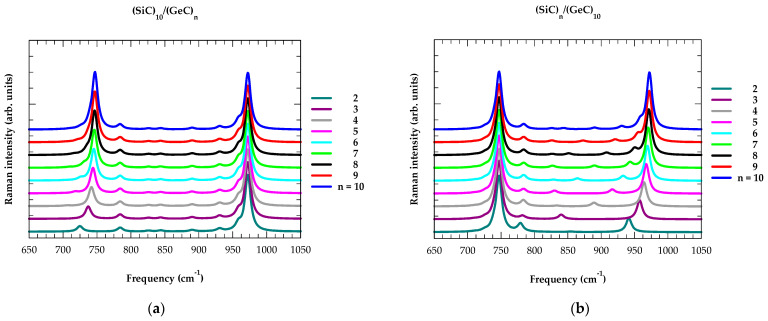

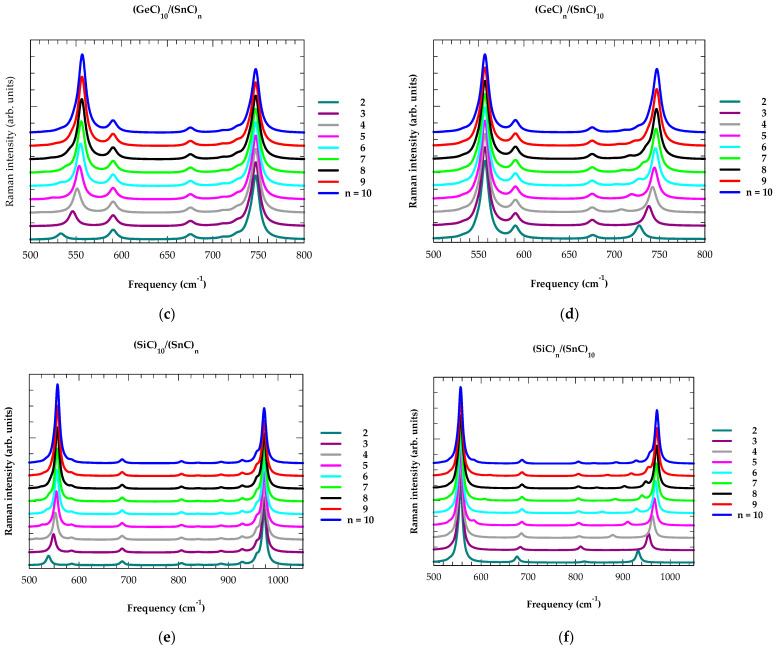
Calculated Raman intensity profiles for the novel C-based (XC)_m_/(YC)_n_ SLs. The simulations were performed using M-LCM and bond-polarizability models. The calculated results reported here are for (**a**) (SiC)_10_/(GeC)_n_, (**b**) (SiC)_n_/(GeC)_10_, *(***c**) (SiC)_10_/(GeC)_n_, (**d**) (SiC)_n_/(GeC)_10_, and (**e**) (SiC)_10_/(GeC)_n_ (**f**) (SiC)_n_/(GeC)_10_ SLs with n = 2, 3, 4, 5, 6, 7, 8, 9, 10 (see: text).

Raman intensity profiles are calculated by meticulously incorporating the interfacial layer thickness ∆ and varying its value in steps from 0 to 3 MLs. First, we present our calculated results (see: Figure 4a–f) for the ideal (XC)_m_/(YC)_n_ SLs with sharp interfaces ∆ = 0. By using an M-LCM, we reported results of phonon dispersions $\omega_{j}^{SL}(\vec{q})$ for the ideal SLs by considering m = n = 10, N = m + n in (XC)_m_/(YC)_n_. This choice has resulted in 20 optical and 20 acoustic phonon branches, which helped us retrieve the high-frequency confined optical phonon modes in the XC- and/or YC-layers. By incorporating the eigenvalues and eigenvectors in the bond polarizability model, we simulated the Raman intensity profiles for (SiC)_m_/(GeC)_n_, (GeC)_m_/(SnC)_n_ and (SiC)_m_/ (SnC)_n_ SLs by using m (n) ≡ 10 and varying the values of n (m) ≡ 2, 3, 4, 5, 6, 7, 8, 9, 10.

The perusal of Raman intensity results displayed in Figure 4a–f for different SLs offered three important features: (a) In (SiC)_10_/(GeC)_10_ SL (see Figure 4a,b), there appeared to be two dominant Raman intensity peaks near the phonon frequencies ω = 972 cm^−1^ (mode = 40) and at ω = 747 cm^−1^ (mode = 22 + 23), respectively. Interestingly, these features were comparable to those of the bulk 3C-SiC and zb GeC $\omega_{LO}$ phonon frequencies. (b) The five lowest phonon frequency modes falling between 17 and 21 were not confined (cf. Section 3.1.2) to the GeC layer in the (SiC)_10_/(GeC)_10_ SL—their dispersions $\omega_{j}^{SL}(\vec{q})$ exhibited bulk-like phonon characteristics. (c) The simulated SiC- [GeC-] like Raman intensity features in (SiC)_10_/(GeC)_n_ [(SiC)_n_/(GeC)_10_] SLs were nearly unaffected, while the GeC (SiC) like intensities consistently shifted upward (downward) as the values of n ≡ 2, 3, 4, 5, 6, 7, 8, 9, 10 changed (possibly due to strain). These observations noticed in (SiC)_m_/(GeC)_n_ SLs were equally applicable to the other (GeC)_m_/(SnC)_n_ (see: Figure 4c,d) and (SiC)_m_/(SnC)_n_ (see: Figure 4e,f) SLs. It should be noted that our Raman intensity profiles for (XC)_m_/(YC)_n_ SLs are in very good agreement with the existing experimental results [150,151] on conventional GaAs/AlAs SLs. 

#### 3.1.6. Raman Intensity Profiles in Graded Superlattices 

**Figure 5:**
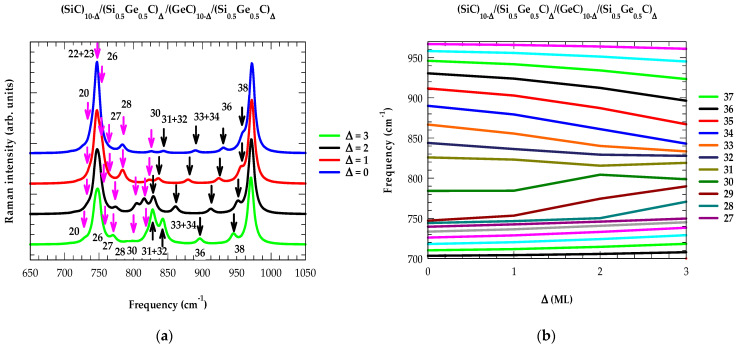

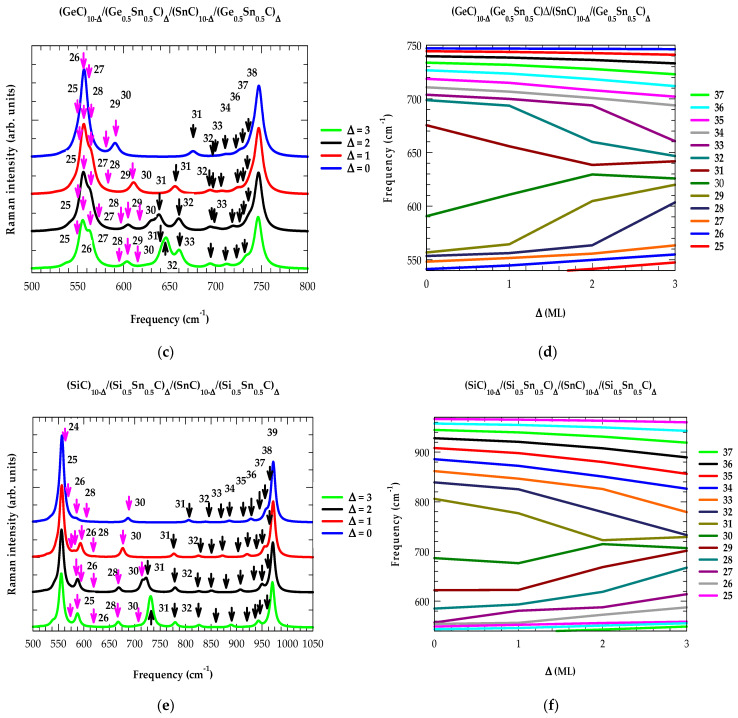
Calculated Raman intensity profiles and the impact of interfacial layer thickness Δ (≡0, 1, 2, 3) on the confined optical phonon frequency shifts in the graded ${\left(XC\right)}_{10-∆}/{\left(X_{0.5}Y_{0.5}C\right)}_{∆}/{\left(YC\right)}_{10-∆}/{\left(X_{0.5}Y_{0.5}C\right)}_{∆}$ SLs. The simulations were performed based on the M-LCM and bond polarizability models. The results reported here are for (**a**,**b**) ${\left(SiC\right)}_{10-∆}/{\left({Si}_{0.5}{Ge}_{0.5}C\right)}_{∆}/{\left(GeC\right)}_{10-∆}/{\left({Si}_{0.5}{Ge}_{0.5}C\right)}_{∆}$, (**c**,**d**) ${\left(GeC\right)}_{10-∆}/{\left({Ge}_{0.5}{Sn}_{0.5}C\right)}_{∆}/{\left(SnC\right)}_{10-∆}/{\left({Ge}_{0.5}{Sn}_{0.5}C\right)}_{∆}$, and (**e**,**f**) ${\left(SiC\right)}_{10-∆}/{\left({Si}_{0.5}{Sn}_{0.5}C\right)}_{∆}/{\left(SnC\right)}_{10-∆}/{\left({Si}_{0.5}{Sn}_{0.5}C\right)}_{∆}$, SLs (see: text).

In Figure 5a–f, we display our calculated results for Raman intensity profiles and interfacial thickness dependent frequency shifts Δ (≡0–3 ML) of the confined optical phonons near the center of m-BZ ($q_{SL}\sim 0$) for graded ${\left(XC\right)}_{10-∆}/{\left(X_{0.5}Y_{0.5}C\right)}_{∆}/{\left(YC\right)}_{10-∆}/{\left(X_{0.5}Y_{0.5}C\right)}_{∆}$ SLs. Identification of different optical phonon modes contributing to the Raman intensity features was carefully made by comparing the calculated SL phonon dispersions $\omega_{j}^{SL}(\vec{q})$ using the M-LCM scheme (cf. Section 3.1.2). In each SL, the impact of interfacial thickness Δ (≡0–3 ML) clearly revealed some interesting features, i.e., (a) the dominant Raman intensity peaks occurring at ω = 972 cm^−1^ (i.e., the mode = 40) and near ω = 747 cm^−1^ (mode = 22 + 23) in ${\left(SiC\right)}_{10-∆}/{\left({Si}_{0.5}{Ge}_{0.5}C\right)}_{∆}/{\left(GeC\right)}_{10-∆}/{\left({Si}_{0.5}{Ge}_{0.5}C\right)}_{∆}$ SLs (see: Figure 5a,b) were comparable to those of the bulk 3C-SiC and zb GeC $\omega_{LO}$ phonon frequencies, and their intensity features remained nearly unchanged with the variation of Δ, (b) the confined optical modes between 34–40 [24,25,26] decreased [increased] almost linearly while the modes between 27–35 caused significant “upward” and “downward” shifts in frequencies by increasing Δ, which contributed meaningful changes to the simulated Raman intensity profiles [see: Figure 5a,b], (c) for Δ = 0, the modes 30–31 in the middle of the optical phonon frequency region were quasi-confined (or IPMs), as their frequencies overlapped with the phonon branches of the neighboring modes, and (d) these phonon modes exhibited atypical trends in the frequency shifts with the increase in Δ. 

Moreover, the phonons became extremely localized and caused significant modifications in the Raman intensity features. The other confined optical phonon modes falling in the range of 24–36 is equally sensitive to the interface broadening Δ and for contributing notable intensity changes due to overlapping of degenerated modes. We strongly feel that (cf. Section 3.1.7) the localization of atomic displacements caused by the optical phonon modes are responsible for triggering atypical frequency shifts which caused the enhancement of calculated Raman intensity features in the graded SLs. Although this interpretation is quite strong, however, it gives only a rough estimation of the interfacial layer thickness Δ causing significant effects on Raman scattering intensities. Moreover, the changes perceived in the simulated Raman line shapes for graded (SiC)_m_/(GeC)_n_, (GeC)_m_/(SnC)_n_, and (SiC)_m_/(SnC)_n_ SLs were found to be consistent with the interpretations made (see: Figure 5a–f, and Table 4) earlier for comprehending Raman scattering results of different strained layer [(Si)_m_/(Ge)_n_, (GaN)_m_ /(AlN)_n_, (InN)_m_/(GaN)_n_, and (InN)_m_/(AlN)_n_] SLs [157,158,159] by using atomic displacement intermixing at the Ge/Si, Ga/Al, In/Ga, and In/Al interfaces. One must note that the estimated atomic displacement effects on the optical phonons for novel C-based graded LDHs are much stronger than those observed previously in different III-V, II-VI and III-N based SLs. 

#### 3.1.7. Atomic Displacements in Superlattices

**Figure 6:**
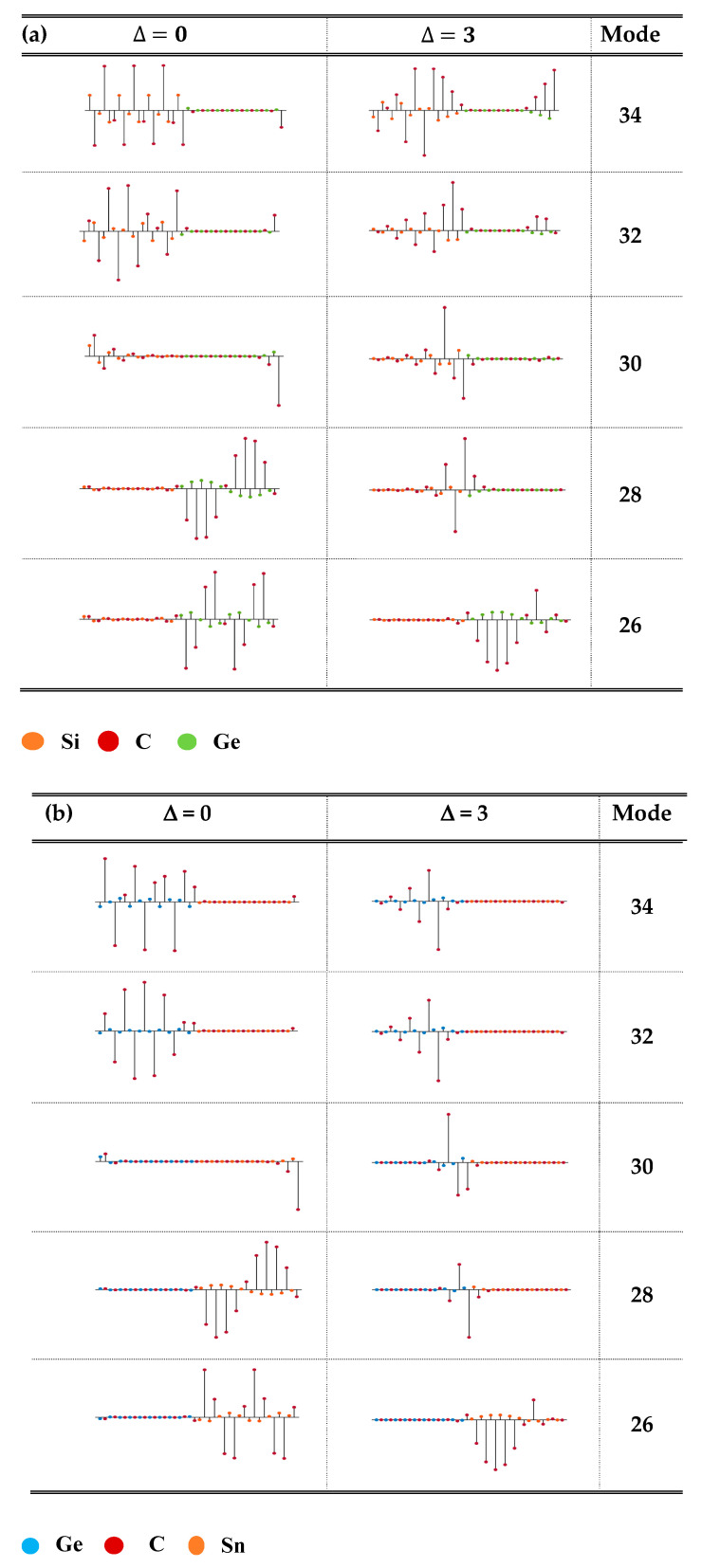

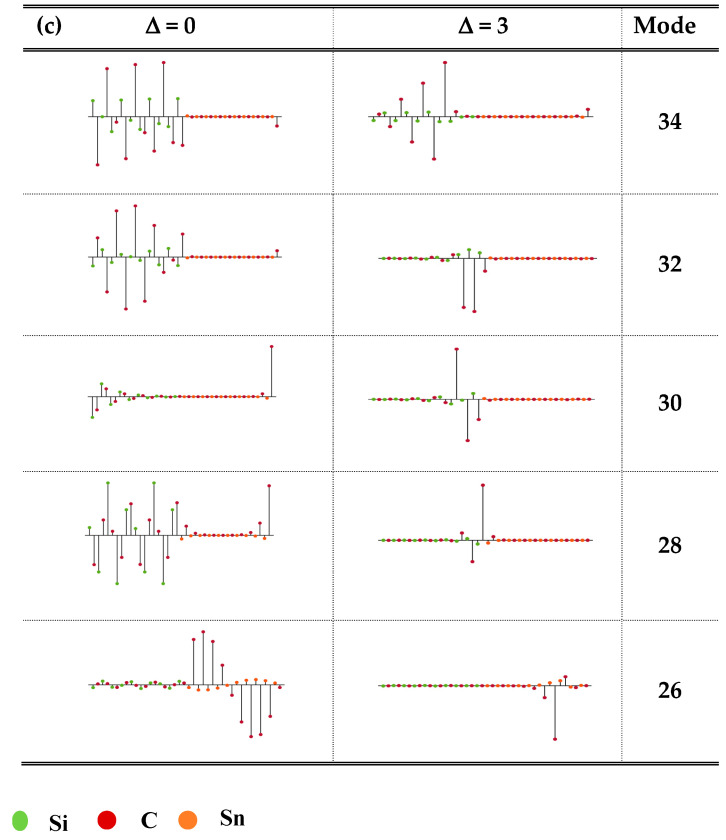
Simulated atomic displacements of selected optical phonon modes that supported the enhancement of calculated Raman intensity profiles: (**a**) ${\left(SiC\right)}_{10-∆}/{\left({Si}_{0.5}{Ge}_{0.5}C\right)}_{∆}/{\left(GeC\right)}_{10-∆}/{\left({Si}_{0.5}{Ge}_{0.5}C\right)}_{∆}$ SL; (**b**) ${\left(GeC\right)}_{10-∆}/{\left({Ge}_{0.5}{Sn}_{0.5}C\right)}_{∆}/{\left(SnC\right)}_{10-∆}/{\left({Ge}_{0.5}{Sn}_{0.5}C\right)}_{∆}$ SL; and (**c**) ${\left(SiC\right)}_{10-∆}/{\left({Si}_{0.5}{Sn}_{0.5}C\right)}_{∆}/{\left(SnC\right)}_{10-∆}/{\left({Si}_{0.5}{Sn}_{0.5}C\right)}_{∆}$ SL. Different colored circles are used to represent the Si, Ge, Sn, and C atoms (see: text).

By incorporating the M-LCM (cf. Section 3.1.2) method, we calculated the atomic displacements in the graded $\;{\left(XC\right)}_{10-∆}/{\left(X_{0.5}Y_{0.5}C\right)}_{∆}/{\left(YC\right)}_{10-∆}/{\left(X_{0.5}Y_{0.5}C\right)}_{∆}$ SLs for all the vibrational modes by choosing different values of Δ (≡0, 1, 2, 3 MLs). In Figure 6a–c, the results of atomic displacements are displayed for a few selected optical modes in three different graded SLs by using the extreme values of Δ ≡ 0 and 3. In ${\left(SiC\right)}_{10-∆}/{\left({Si}_{0.5}{Ge}_{0.5}C\right)}_{∆}/{\left(GeC\right)}_{10-∆}/{\left({Si}_{0.5}{Ge}_{0.5}C\right)}_{∆}$ SL (for instance), the lowest folded acoustic modes 1–13 with ω < 350 cm^−1^ were found propagating through the entire SL. Varying the interfacial layer thickness Δ (≡1, 2, 3) caused no appreciable changes in the 1–13 (cf. Figure 3b) modes. Other acoustic phonons related to the modes 14–20 with frequencies (350–630 cm^−1^) exhibited non-dispersive characteristics, usually perceived for the optical modes—their atomic vibrations were confined in the SiC layers. In an ideal situation (Δ ≡ 0), the high-frequency longitudinal optical modes 21–22 would be extended, while the other optical phonons exhibited confinement characteristics falling either in the GeC- or in the SiC-layers. 

Most importantly, the COMs in C-based SLs are shown to have high sensitivity on the interfacial layer thickness for causing significant changes in phonon frequencies by increasing the values of Δ from 0 to 3 MLs. Obviously, there were modes which instigated either new or enhanced Raman intensities due to the overlapping of nearly degenerated optical phonon frequencies. Clearly, this intuition is completely supported by our calculated results of large $∆\omega_{LO}$ energy shifts in the phonon frequency region of 800–850 cm^−1^. For Δ ≡ 3 we have noticed substantial enhancement of Raman spectral features for ${\left(SiC\right)}_{10-∆}/{\left({Si}_{0.5}{Ge}_{0.5}C\right)}_{∆}/{\left(GeC\right)}_{10-∆}/{\left({Si}_{0.5}{Ge}_{0.5}C\right)}_{∆}$ SLs (see: Figure 5a,b). The perusal of our simulated atomic displacements (see: Figure 6a) revealed the highest value of interfacial layer thickness, triggering significant changes in the optical phonon modes from confined to the localized one. Similar features are also perceived in ${\left(GeC\right)}_{10-∆}/{\left({Ge}_{0.5}{Sn}_{0.5}C\right)}_{∆}/{\left(SnC\right)}_{10-∆}/{\left({Ge}_{0.5}{Sn}_{0.5}C\right)}_{∆}$ (see: Figure 5c,d and Figure 6b) and ${\left(SiC\right)}_{10-∆}/{\left({Si}_{0.5}{Sn}_{0.5}C\right)}_{∆}/{\left(SnC\right)}_{10-∆}/{\left({Si}_{0.5}{Sn}_{0.5}C\right)}_{∆}$ (see: Figure 5e,f and Figure 6c) SLs. These observations have corroborated our reasonings for causing major enhancement of the Raman intensity profiles. 

## 4. Conclusions

In conclusion, we reported the results of our systematic study for comprehending the FAPs, COMs, and IPMs in novel C-based SLs using Rytov, M-LCM, and elasticity models. In the absence of experimental Raman scattering spectroscopy results and reliable theoretical data on SLs, it is difficult to assess the accuracy of simulated characteristics for the phonon dispersions $\omega_{j}^{SL}(\vec{q})$ of the (XC)_m_/(YC)_n_ materials. However, we linked our predictions of atypical phonon characteristics (viz. propagating optical, acoustic, and confined modes) in XC/YC superlattices to the large differences in masses of X,Y anions and common C cation atoms. While the projections of phonon modes for the (XC)_m_/(YC)_n_ SLs were in striking contrast with the traditional GaAs/AlAs SLs, the calculations of $\;\omega_{j}^{SL}(\vec{q})$ emerged well within the range of the phonon dispersions of individual XC-YC materials. Interfacing effects on the acoustic and optical phonons in the graded ${\left(XC\right)}_{10-∆}/{\left(X_{0.5}Y_{0.5}C\right)}_{∆}/{\left(YC\right)}_{10-∆}/{\left(X_{0.5}Y_{0.5}C\right)}_{∆}$ SLs were carefully studied. Calculated FAPs using M-LCM showed negligible effects on the interfacial thickness Δ. However, COMs revealed significant “downward” and/or “upward” shifts in frequencies by increasing Δ. Consequently, our study indicated overlapping modes, which either caused new and/or enhanced Raman intensity features in the optical phonon frequency region. The simulated Raman intensity profiles provided a very good agreement with the earlier experimental reports on GaN/Al_x_Ga_1−x_N SLs [155,156], corroborating the presence of a graded alloy interface with a thickness of nearly ~2 nm. Assessment of the overall interfacing effects in (XC)_m_/(YC)_n_ SLs leads us to believe that the optical phonons can be used as a probe to appraise the interfacial broadening that caused considerable shifts in phonon frequencies and triggered increase in the Raman intensity profiles. Therefore, we strongly feel that our systematic findings on the phonon characteristics for the novel (XC)_m_/(YC)_n_ SLs will encourage Raman spectroscopists [150,157,158,160] to perform similar measurements to check our theoretical conjectures. Such measurements are also expected to provide the necessary parameters for regulating and optimizing the epitaxial growth processes required for achieving C-based structures to develop the NS devices for different types of applications. 

The study of heat conduction in LDHs is a comprehensive and very challenging issue. Understanding the mechanisms controlling thermal processes in SLs requires correct phonons [160,161,162,163,164,165,166] along the growth as well as in-plane directions. These phonons in SLs must be obtained accurately by using realistic lattice dynamical models. One must note that the degeneracies of transverse [$\omega_{TO}\;\left(\omega_{TA}\;\right)]$ modes $\;in\;\omega_{j}^{SL}\left(\vec{q}\right)$ are lifted in the in-plane direction. Due to spatial confinement and zone-folding effects, the SL optical (acoustical) phonons are expected to exhibit a strong mixture of the $\omega_{TO}$, $\omega_{LO}$ $(\omega_{TA}$, $\omega_{LA})$ GeC-like, and SiC-like modes. Our recent study [167] revealed complicated phonon dispersion curves with the appearance of several acoustic stop bands at certain finite values of wavevectors $\vec{\;q}$. In earlier studies, such SL stop bands for the acoustic phonons were observed experimentally at oblique incidences and studied theoretically by using an elastic theory. These modifications of phonon dispersions $\omega_{j}^{SL}\left(\vec{q}\right)$ are expected to have a direct impact on the acoustic phonon properties of NS materials, including the phonon group velocity, density of states, and thermal conductivity [148,161,162,163,164,165,166]. Besides thermal transport in the growth direction, the in-plane thermal transport is equally valuable for electro-optic and thermoelectric applications, as these characteristics can trigger significant effects when studying thermal management applications. Earlier calculations of effective thermal conductivity in the conventional GaAs/AlAs SLs for in-plane and cross-plane directions [148] exhibited results which are different from the experimental data. Reliable experimental measurements and accurate simulations of $\omega_{j}^{SL}\left(\vec{q}\right)$ are very much needed for examining the polarization and period-dependent thermal conductivity not only in C-based materials but also in many other technologically important SLs.

## Figures and Tables

**Table 3:** Calculated strain-induced shift in the optical ($\omega_{LO},\;\omega_{TO}$) phonon frequencies (cm^−1^) using a classical elastic model for (A) (SiC)_m_/(GeC)_n_, (B) (GeC)_m_/(SnC)_n_, and (C) (SiC)_m_/(SnC)_n_ superlattices. The in-plane and perpendicular strain parameters at the material interface are also given (see text).

(A) (SiC)_m_/(GeC)_n_								
m/n	$∆\omega_{LO}^{SiC}$	$∆\omega_{TO}^{SiC}$	$∆\omega_{LO}^{GeC}$	$∆\omega_{TO}^{GeC}$	$\epsilon_{\left|\right|}^{SiC}$	$\epsilon_{⊥}^{SiC}$	$\epsilon_{\left|\right|}^{GeC}$	$\epsilon_{⊥}^{GeC}$
8/2	−16.1	−0.565	44.9	1.71	0.0103	−0.0075	−0.0403	0.0275
8/4	−26.9	−0.945	37.6	1.43	0.0173	−0.0126	−0.0337	0.0230
8/6	−34.6	−1.22	32.3	1.23	0.0223	−0.0162	−0.0289	0.0197
8/8	−40.5	−1.42	28.3	1.08	0.0260	−0.0190	−0.0254	0.0173
8/10	−45.0	−1.58	25.2	0.959	0.0290	−0.0211	−0.0226	0.0154
10/8	−35.9	−1.26	31.4	1.20	0.0231	−0.0168	−0.0282	0.0192
10/6	−30.2	−1.06	35.3	1.34	0.0195	−0.0142	−0.0316	0.0215
10/4	−23.0	−0.809	40.2	1.53	0.0148	−0.0108	−0.0361	0.0246
10/2	−13.4	−0.470	46.8	1.78	0.0086	−0.0063	−0.0419	0.0286
**(B) (GeC)_m_/(SnC)_n_**								
**m/n**	$∆\omega_{LO}^{GeC}$	$∆\omega_{TO}^{GeC}$	$∆\omega_{LO}^{SnC}$	$∆\omega_{TO}^{SnC}$	$\epsilon_{\left|\right|}^{GeC}$	$\epsilon_{⊥}^{GeC}$	$\epsilon_{\left|\right|}^{SnC}$	$\epsilon_{⊥}^{SnC}$
8/2	−21.8	−0.928	65.4	5.20	0.0178	−0.0121	−0.0894	0.0821
8/4	−38.2	−1.61	56.8	4.52	0.0309	−0.0210	−0.0777	0.0713
8/6	−50.3	−2.14	50.2	4.00	0.0409	−0.0279	−0.0687	0.0631
8/8	−60.1	−2.55	45.0	3.58	0.0489	−0.0333	−0.0615	0.0565
8/10	−68.1	−2.89	40.8	3.24	0.0554	−0.0377	−0.0557	0.0512
10/8	−52.5	−2.23	49.1	3.91	0.0427	−0.0291	−0.0671	0.0616
10/6	−43.3	−1.84	54.0	4.30	0.0352	−0.0240	−0.0738	0.0678
10/4	−32.0	−1.36	60.0	4.77	0.0260	−0.0178	−0.0820	0.0753
10/2	−18.0	−0.765	67.4	5.36	0.0146	−0.0100	−0.0922	0.0874
**(C) (SiC)_m_/(SnC)_n_**								
**m/n**	$∆\omega_{LO}^{SiC}$	$∆\omega_{TO}^{SiC}$	$∆\omega_{LO}^{SnC}$	$∆\omega_{TO}^{SnC}$	$\epsilon_{\left|\right|}^{SiC}$	$\epsilon_{⊥}^{SiC}$	$\epsilon_{\left|\right|}^{SnC}$	$\epsilon_{⊥}^{SnC}$
8/2	−40.6	−1.430	93.6	7.45	0.0261	−0.0190	−0.128	0.118
8/4	−70.7	−2.49	81.5	6.49	0.0454	−0.0331	−0.111	0.102
8/6	−94.0	−3.31	72.2	5.75	0.0604	−0.0440	−0.099	0.091
8/8	−113.0	−3.96	64.9	5.16	0.0723	−0.0526	−0.089	0.081
8/10	−128.0	−4.49	58.8	4.68	0.0820	−0.0597	−0.080	0.074
10/8	−98.1	−3.45	70.6	5.62	0.0630	−0.0459	−0.097	0.089
10/6	−80.7	−2.84	77.6	6.17	0.0519	−0.0378	−0.106	0.097
10/4	−59.7	−2.10	86.0	6.84	0.0383	−0.0279	−0.118	0.108
10/2	−33.5	−1.18	96.4	7.67	0.0215	−0.0157	−0.132	0.121

**Table 4:** The calculated shifts of $∆\omega_{o}$ confined optical phonon modes based on modified linear-chain model. These shifts of phonon modes between 27 and 35 are obtained at the zone center of superlattice m-BZ ($q_{SL}=0$) as the interfacial layer thickness Δ is varied between 0, 1, 2, 3 ML. All the phonon values are in cm^−1^.

$(A)\;\;\;\;\;\;\;\;\;\;\;\;\;\;\;\;\;\;\;\;\;\;\;\;\;\;\;{\left(SiC\right)}_{10-∆}/{\left({Si}_{0.5}{Ge}_{0.5}C\right)}_{∆}/{\left(GeC\right)}_{10-∆}/{\left({Si}_{0.5}{Ge}_{0.5}C\right)}_{∆}$				
	$\omega_{o};∆=0$	$∆\omega_{o};∆=1$	$∆\omega_{o};∆=2$	$∆\omega_{o};∆=3$
35	911.7	−8.85	−24.54	−44.59
34	890.1	−10.8	−29.04	−47.2
33	866.7	−11.13	−26.6	−33.37
32	843.8	−7.57	−14.59	−16.05
31	825.9	−2.77	−10.35	−6.84
30	784.09	0.19	20.2	14.56
29	747.03	6.33	27.37	42.84
28	744.16	2.4	6.05	26.53
27	739.52	2.93	6.31	10.11
** $(B)\;\;\;\;\;\;\;\;\;\;\;\;\;\;\;\;\;\;\;\;\;\;\;\;\;\;\;\;{\left(GeC\right)}_{10-∆}/{\left({Ge}_{0.5}{Sn}_{0.5}C\right)}_{∆}/{\left(SnC\right)}_{10-∆}/{\left({Ge}_{0.5}{Sn}_{0.5}C\right)}_{∆}$ **				
	$\omega_{o};∆=0$	$∆\omega_{o};∆=1$	$∆\omega_{o};∆=2$	$∆\omega_{o};∆=3$
35	718.7	−3.8	−10.6	−16.6
34	710.8	−4.2	−9.96	−16.9
33	703.8	−3.84	−9.92	−43.3
32	698.8	−5.2	−38.9	−52.2
31	675.4	−19.6	−36.96	−33.6
30	590.7	20.1	38.9	35.1
29	556.9	7.7	47.9	63.1
28	553.6	2.7	10.0	49.9
27	548.3	3.4	7.5	15.2
** $(C)\;\;\;\;\;\;\;\;\;\;\;\;\;\;\;\;\;\;\;\;\;\;\;\;\;\;\;\;{\left(SiC\right)}_{10-∆}/{\left({Si}_{0.5}{Sn}_{0.5}C\right)}_{∆}/{\left(SnC\right)}_{10-∆}/{\left({Si}_{0.5}{Sn}_{0.5}C\right)}_{∆}$ **				
	$\omega_{o};∆=0$	$∆\omega_{o};\;∆=1$	$∆\omega_{o};\;∆=2$	$∆\omega_{o};\;∆=3$
35	908.3	−10.4	−28.3	−52.0
34	885.7	−13.3	−34.9	−59.4
33	861.6	−15.1	−35.9	−82.3
32	839.2	−14.0	−59.9	−106.4
31	806.0	−29.1	−83.3	−76.9
30	686.7	−10.3	28.1	20.1
29	622.1	0.5	46.5	79.4
28	585.2	8.3	33.7	82.3
27	557.0	23.7	30.8	57.1

## Data Availability

The original contributions presented in the study are included in the article, further inquiries can be directed to the corresponding authors.
